# Fulvestrant May Falsely Increase 17β-Estradiol Levels in Immunoassays: A Case Report of a 57-Year-Old Postmenopausal Patient With Recurrent Estrogen Receptor-Positive Breast Cancer

**DOI:** 10.3389/fonc.2022.832763

**Published:** 2022-04-13

**Authors:** Jingxian Ding, Yali Cao, Yonghong Guo

**Affiliations:** ^1^Department of Radiation Oncology, The Breast Cancer Institute, Third Hospital of Nanchang, Nanchang, China; ^2^Department of Breast Surgery, The Breast Cancer Institute, The Third Hospital of Nanchang, Nanchang, China; ^3^Department of Radiation Oncology, The Fourth Affiliated Hospital of Nanchang University, Nanchang, China

**Keywords:** breast cancer, antihormonal therapy, reirradiation (re-RT), 17β-estradiol (E2), fulvestrant

## Abstract

The prognosis for female patients with locoregionally recurrent breast cancer has improved with the concurrent local and systemic treatment under multiple disciplinary teams. Radiotherapy is a valuable local treatment measure for unresectable locoregional recurrent breast cancer; however, reirradiation in previously irradiated areas is still a matter of debate. Antihormonal therapy achieves an overall survival benefit for most of these patients with estrogen receptor-positive (ER+) breast cancer in both adjuvant and metastatic settings. Fulvestrant is an ER antagonist and selective ER downregulator widely used in antihormonal therapy, especially in recurrent postmenopausal ER+ breast cancers. However, fulvestrant closely resembles 17β-estradiol in its molecular structure which may result in false increases in serum 17β-estradiol levels in commercially available immunoassays leading to incorrect medical decisions. Herein, we report a case of a 57-year-old postmenopausal patient with recurrent ER+ breast cancer treated with concurrent fulvestrant and reirradiation. There was a good clinical response, and the combination treatment was well tolerable. During the quarterly follow-up, we monitored a gradual increase of the serum 17β-estradiol level in immunoassays, unexpectedly, because the patient underwent natural menopause 8 years ago. To rule out the suspected fulvestrant cross-reactivity with 17β-estradiol in immunoassay, the patient’s serum 17β-estradiol levels were subsequently tested with the more sensitive and specific liquid chromatography-mass spectrometry (LC-MS) method, which confirmed 17β-estradiol levels at the postmenopausal level. Concomitant fulvestrant with reirradiation seems to be a safe and effective therapy for locoregionally recurrent ER+ breast cancer. However, a falsely increased 17β-estradiol may result from cross-reactivity between 17β-estradiol and its molecular analog compounds, for example, fulvestrant. Therefore, it is important for the clinicians with the knowledge of this interaction to prevent unnecessary erroneous interpretation of results and avoid wrong medical decisions.

## Introduction

Breast cancer is one of the most common malignant tumors in women and the leading cause of cancer-related death, approximately 70% to 80% of which are estrogen receptor-positive (ER+) (1, 2). Since the policy of breast cancer screening and advances in adjuvant local and systemic treatment, both recurrence and mortality rates of breast cancer have decreased steadily and 5-year overall survival currently exceeds 90% in some countries (3). With a gradual improvement of primary breast cancer treatment, including more extensive use of antihormonal therapy, improved cancer control over time has been achieved. Antihormonal therapy achieves an overall survival benefit for most of these patients in both adjuvant and metastatic settings (4, 5). However, because of the incidence of breast cancer rising and mortality rates declining, there are an increasing number of breast cancer survivors, and the accumulating number of patients with treatment failure is large. The locoregional recurrence of breast cancer is a well-known independent prognostic factor for distant metastasis and poor survival. Treatment for the locoregional recurrence of breast cancer has evolved during the last decades, and a multidisciplinary approach is presently strongly considered (6). Whenever possible, it should be treated with curative intent, preferably with complete excision, otherwise with biopsy and local and/or systemic therapy as indicated. Unresectable locoregional recurrent breast cancer in previously irradiated areas is a life-threatening disease, and optimal treatment is still a matter of debate. Reirradiation may be a valid local treatment option with caution for selected cases (7, 8). Endocrine treatment should be offered to all patients with estrogen receptor-positive (ER+) breast cancers (4, 9, 10). Fulvestrant is a pure estrogen receptor antagonist and selective estrogen receptor downregulator widely used in antihormonal therapy, especially in recurrent postmenopausal ER+ breast cancers (10–12).

However, fulvestrant closely resembles 17β-estradiol in its molecular structure which may result in a false increase in serum 17β-estradiol levels in commercial immunoassays leading to incorrect medical decisions (10–12). Herein, we report an interesting case of a 57-year-old postmenopausal patient with recurrent ER+ breast cancer treated with concomitant reirradiation and fulvestrant. There was a good clinical response to this combination treatment.

During the course of the follow-up, we monitored a gradually increased serum 17β-estradiol level in immunoassay. This finding was puzzling because the patient reported menopausal 8 years ago. To further clarify the unexpectedly increased 17β-estradiol level, the patient’s serum 17β-estradiol levels were subsequently obtained and tested with a more sensitive and specific liquid chromatography–mass spectrometry (LC-MS) method (13, 14). The final result confirmed the patient’s serum 17β-estradiol levels at the postmenopausal level. The purpose of the case report is to remind medical colleagues about the possibility of falsely increased 17β-estradiol levels in patients treated with fulvestrant.

## Case Presentation

**Figure 1:**
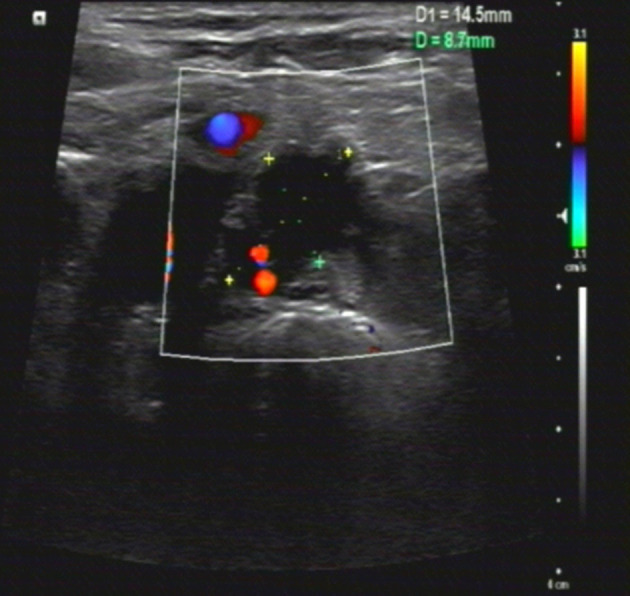
Ultrasonography features of the case. Ultrasonic images showed a 1.5 * 0.9CM hypoechoic nodule in the left supraclavicular area, with abundant blood flow signals, which indicated cancer metastatic lymph nodes.

**Figure 2:**
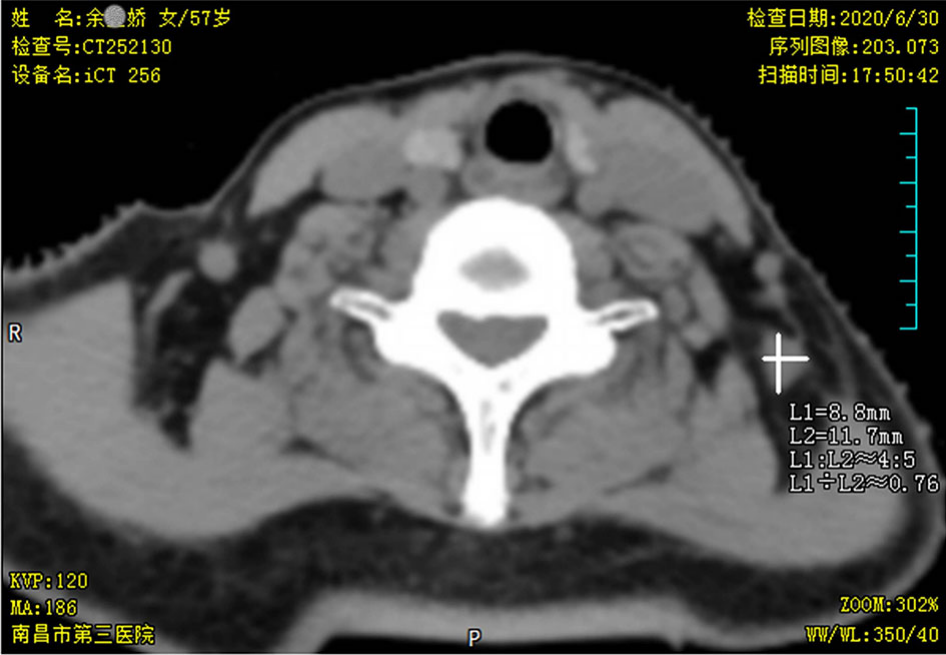
Neck computed tomography (CT) scan findings. Pretreatment axial neck computed tomography (CT) slice taken in the region of supraclavicular fossa revealed a 1.2 * 0.9-cm soft tissue mass in nodal level V.

In June 2020, after a disease-free interval of about 7 years, during the course of follow-up, a 57-year-old patient was first found with supraclavicular adenopathy by ultrasonography (**Figure 1**), and computed tomography (CT) scan showed a 1.2 * 0.9-cm lymph node apparently strengthened with contrast (**Figure 2**). Core needle biopsy confirmed a metastasis of breast adenocarcinoma with the presence of both ER and progesterone receptors (PR) in 90% of tumor cells with strong staining, HER-2 2+ with no amplification in fluorescent *in situ* hybridization (FISH), and Ki67 positive in 40% tumor cells. We offered a thorough examination to exclude any distant metastases. Laboratory exams were unremarkable before commencement of concurrent fulvestrant with reirradiation treatment, including tumor markers and a reproductive hormone profile. Serum levels of AFP, CEA, CA125, and CA153 tumor markers were 4.00 ng/ml, 1.03 ng/ml, 14.90 U/ml, and 6.60 U/ml, respectively (**Table 1**). The serum levels of 17β-estradiol (E2), follicle-stimulating hormone (FSH), and luteinizing hormone (LH) were 11.94 (pg/ml), 9.65 (mIU/ml), and 3.22 (mIU/ml) (**Table 2**). The rest of the physical examination was also all within normal limits.

**Table 1:** The concentration variations of the patient’s serum tumor markers during antihormonal therapy in immunoassay.

Test time	AFP (ng/ml)	CEA (ng/ml)	CA-125 (U/ml)	CA-153 (U/ml)
**2021/10/8 11:11**	3.60	1.15	11.70	10.30
**2021/7/8 9:09**	3.70	1.53	9.70	7.30
**2021/4/21 9:08**	1.80	1.54	11.40	6.50
**2020/12/22 9:18**	3.40	1.05	11.80	6.30
**2020/9/22 11:05**	3.20	1.08	11.80	8.10
**2020/6/11 9:08**	3.00	0.82	12.20	6.10
**2020/6/5 9:39**	4.00	1.03	14.90	6.60

Reference normal range in immunoassay. AFP (alpha fetoprotein) 0–8.1 ng/ml, CEA (carcinoembryonic antigen) 0–10 ng/ml, CA-125 0–30.2 U/ml, CA-153 0–32.4 U/ml.

**Table 2:** The concentration variations of the patient’s serum gonadal hormone during antihormonal therapy in immunoassay.

Test time	E2 (pg/ml)*^a^*	FSH (mIU/ml)*^b^*	LH (mIU/ml)*^c^*
**2021/10/8 11:11**	263.94	64.39	18.96
**2021/7/8 9:09**	317.82	62.70	26.23
**2021/4/21 9:09**	174.79	68.18	30.97
**2020/9/22 11:04**	123.32	41.54	20.45
**2020/6/11 9:08**	11.94	9.65	3.22

E2, 17β-estradiol; FSH, follicle-stimulating hormone; LH, luteinizing hormone.

Reference range in immunoassay (female).

^a^Follicular stage 19.5–144 pg/ml, mid-cycle stage 64.0–357.0 pg/ml, luteal stage 56.0–214.0 pg/ml, postmenopausal stage 23.0–116.3 pg/ml.

^b^Follicular stage 2.5–10.2 mIU/Ml, mid-cycle stage 3.4–33.4 mIU/ml, luteal stage 1.5–9.1 mIU/ml, postmenopausal stage 23.0–116.3 mIU/ml.

^c^Follicular stage 2.0–13.0 mIU/Ml, mid-cycle stage 8.0–76.0 mIU/ml, luteal stage 1.0–17.0 mIU/ml, postmenopausal stage 16.0–54.0 mIU/ml.

In June 2013, the patient underwent modified radical mastectomy for the diagnosis of infiltrating ductal carcinoma (IDC) of the left breast. The pathology revealed IDC, moderately differentiated (G2), measuring 3.5 * 2 * 1.5 cm. There was 1 of 18 lymph nodes that had macrometastatic disease. Immunohistochemical (IHC) examination showed the presence of both ER and PR in 80% of tumor cells with strong staining, while HER-2 showed negative, and Ki67 positive, in 20% tumor cells. The pathologic stage was determined to be pT2N1 (stage II) according to the AJCC staging system. The patient received postoperative adjuvant chemotherapy consisting of 4 cycles of triweekly epirubicin and cyclophosphamide, then 4 cycles of triweekly paclitaxel followed between June 2013 and December 2013. She also received postmastectomy radiotherapy (PMRT), with a total dose of 50 Gy at 2 Gy/fraction × 25 fractions to the chest wall and the supra-/infra-clavicular region (SCN), with intensity-modulated radiation therapy (IMRT) and 6-MV photon. PMRT was completed in January 2014. Antihormonal therapy with highly selective non-steroidal aromatase inhibitor anastrozole was given regularly after confirming her postmenopausal status without interruption. The patient completed her 5 years of antihormonal therapy in January 2019.

**Figure 3:**
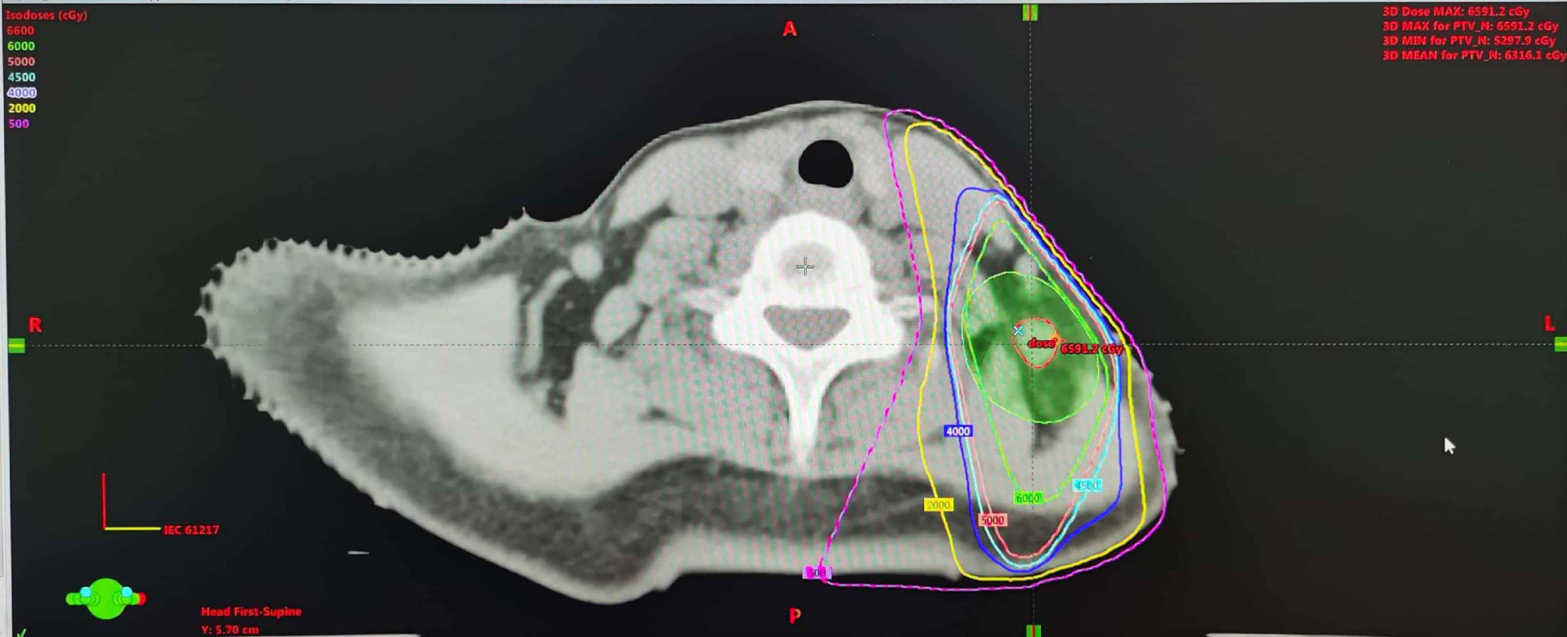
Dose distribution of the treatment plan using 3-dimensional conformal radiation therapy. The dose distribution of the reirradiation plan (30 F × 2 Gy) with 3-dimensional conformal radiation therapy.

**Figure 4:**
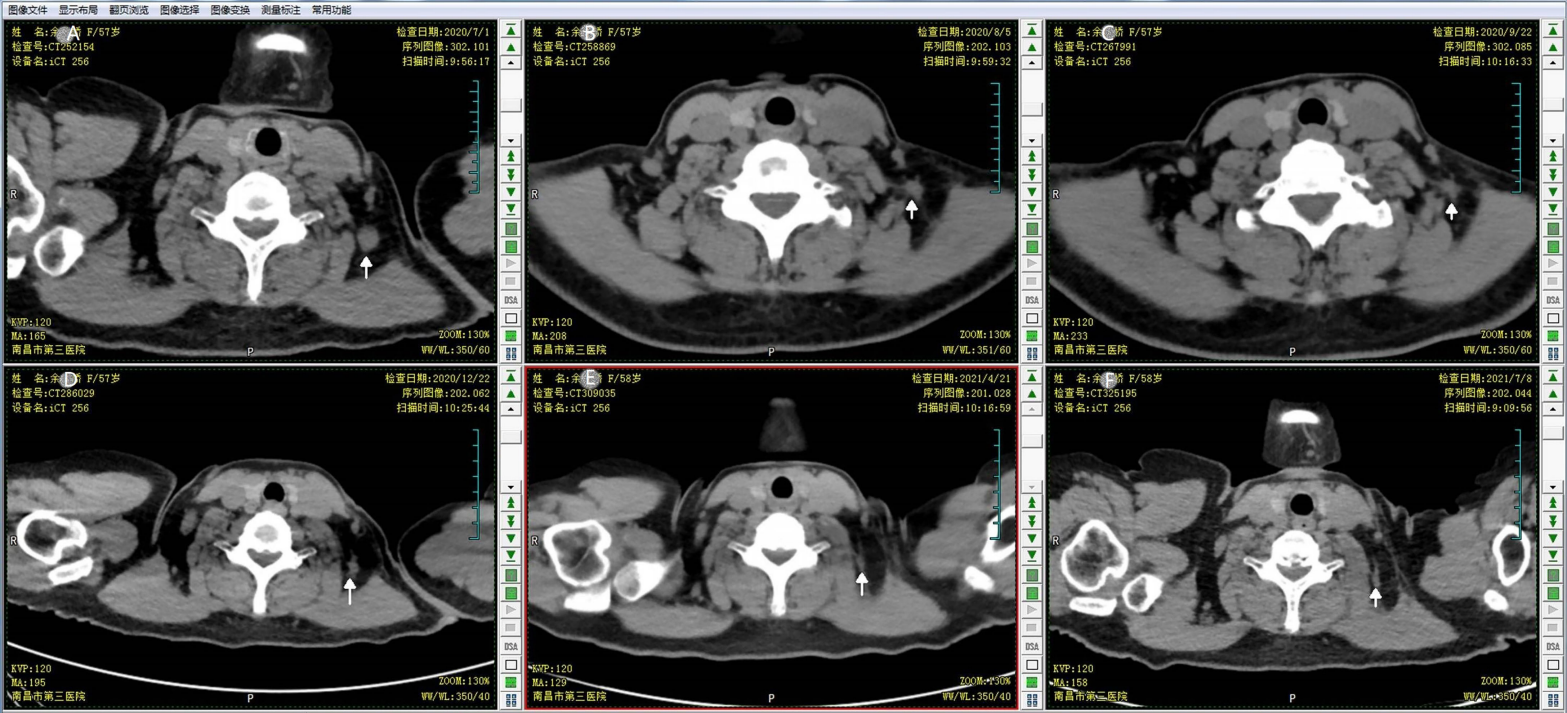
The response of the targeted lymph node to the concomitant fulvestrant with reirradiation. CT scan monitored the continuous response of the breast cancer metastatic neck lymph node during the follow up. **(A)** CT simulation image prior to the first treatment; **(B)** CT scan image at end of the 25th fraction of reirradiation; **(C)** follow-up CT scan at 1 month after the end of reirradiation therapy; **(D)** follow-up CT scan at 3 months after reirradiation therapy; **(E)** follow-up CT at 7 months after reirradiation therapy; **(F)** follow-up CT at 10 months after reirradiation therapy.

For this first locoregional recurrent breast cancer, given the patient refused surgical treatment, concurrent fulvestrant (500 mg intramuscular injection every 4 weeks) with reirradiation as a palliative treatment was proposed under our multiple disciplinary team (MDT). The scheduled radiation dose was 60 Gy in 30 fractions with 6-MV X-ray delivered by 3-dimensional conformal radiation therapy (3D-CRT) conducted to the primary tumor volume (**Figure 3**). The patient had a good clinical response and tolerated well to this combination treatment. At the end of the radiotherapy, CT scan showed a continuously radiologic response of the metastatic lymph node (RECIST 1.1) (**Figure 4**).

The patient still underwent treatment with fulvestrant 500 mg intramuscular injection every 4 weeks and had good quality of life. During the quarterly follow-up, tumor markers remained within normal limits during the course of fulvestrant intramuscular injection (**Table 1**), while the serum 17β-estradiol level was gradually increased unexpectedly after fulvestrant intramuscular injection in immunoassays (**Table 2**). This finding was puzzling because the patient reported menopausal 8 years ago. To further evaluate the unexpectedly increased 17β-estradiol level, the patient’s serum 17β-estradiol levels were subsequently obtained and tested with a more sensitive and specific LC-MS method. The concentration of 17β-estradiol was 5.6 ng/ml indicating the patient’s postmenopausal status in LC-MS, while 263.94 ng/ml in immunoassays, which effectively confirmed that an exogenous compound fulvestrant likely caused the cross reactivity with the immunoassay resulting in falsely increased serum 17β-estradiol readings. The course of the breast cancer patient is summarized in **Table 3**. In the recent follow-up, the patient remained progression-free with fulvestrant treatment.

**Table 3:** The brief course of disease in the patient diagnosed with breast cancer.

Time frame	Line of treatment	Regimen	Response
**June 2013**	Mastectomy	Modified radical mastectomy	Complete response (CR)
**June 2013–Dec 2013**	Adjuvant chemotherapy	Epirubicin + cyclophosphamide × 4 cycles, followed by docetaxel × 4 cycles	
**Dec 2013–Jan 2014**	Adjuvant postmastectomy radiotherapy (PMRT)	Chest wall plus the supra-/infra-clavicular region (SCN) total dose of 50 Gy at 2 Gy/day × 25 fractions	
**Feb 2014–Jan 2019**	Adjuvant antihormonal therapy	Anastrozole	
**June 2020–**	Recurrent supraclavicular lymph node metastatic HER-2-negative luminal B breast cancer	Concomitant fulvestrant with reirradiation, fulvestrant 500 mg intramuscular injection every 4 weeks, reirradiation to the involved lymph node, the total radiation dose was 60 Gy in 30 fractions	Partial response at the end of radiotherapy, clinical complete response (cCR)

## Discussion

Local failure is one of the most frequent events in breast cancer; both surgical resection and radiotherapy are effective treatment measures. However, reirradiation for breast cancer is challenging under certain circumstances. Fulvestrant is an effective treatment option and likely to be increasingly used in postmenopausal women with recurrent hormone receptor-positive breast cancer. Previous reports have demonstrated that fulvestrant radiosensitizes ER+ human breast cancer cells (15–17).

In our daily clinical practice, most serum gonadal hormone measurements are performed in immunochemical assays. The main principle of immunoassays is the detection of an antigen–antibody reaction. A known limitation of gonadal hormone immunoassays is cross-reactivity caused by structural similar compounds to the target hormone of the assay. It is reported that fulvestrant possesses the basic structure of 17β-estradiol, which may result in false positive results in the immunoassay (11, 18, 19, 20).

For this patient with a gradual increase in serum 17β-estradiol levels presented here, during her 17β-estradiol measurements, she was being treated with fulvestrant monthly. This raised doubts as to whether she was really in established menopause or under consideration of the possibility of a false positive result caused by fulvestrant. On the basis of this structural similarity, we believe that fulvestrant was the most possible compound that led to the falsely increased 17β-estradiol level. It is reported that liquid chromatography–mass spectroscopy (LC-MS) offers superior analytical sensitivity and specificity compared to immunochemical methods (13, 14). Therefore, it is applied to monitor aromatase inhibitor treatment of breast cancer patients with the combined use of a luteinizing hormone-releasing hormone (LH-RH) analogue for premenopausal patients. As opposed to immunoassays, LC-MS does not rely on antibodies and therefore avoids false positive results. Consequently, the serum of the patient treated with fulvestrant was concurrently analyzed by both LC-MS and immunoassays. The final result of LC-MS confirmed the patient’s serum 17β-estradiol levels at the postmenopausal level, which indicated that the increase in 17β-estradiol level in immunoassay was due to the fulvestrant cross-reactivity with 17β-estradiol.

After reviewing the available literature, we found that there were quite a few reports on the falsely increased 17β-estradiol levels and the cross-reactivity between antihormonal agents and standard commercially available 17β-estradiol immunoassays. Fulvestrant sharing the basic structure of 17β-estradiol has also scarcely been reported to cause falsely increased 17β-estradiol levels or cross-reactivity with 17β-estradiol immunoassays. The case presented here may help clinicians to differentiate the falsely increased 17β-estradiol levels to avoid wrong medical decisions.

## Conclusion

Concomitant fulvestrant with reirradiation seems to be a safe and effective therapy for unresectable locoregional recurrent ER+ breast cancer. Serum 17β-estradiol levels reflecting menopause status may affect treatment decisions for patients with ER+ breast cancer. However, a falsely increased 17β-estradiol level may result from cross-reactivity between 17β-estradiol and its molecular analog compounds. For patients receiving fulvestrant, spurious results may be generated that could impact treatment decisions, so LC-MS is strongly recommended in this setting. It is also important for the clinicians to know this interaction to prevent unnecessary erroneous interpretation of results and wrong medical decisions.

## Data Availability Statement

The original contributions presented in the study are included in the article. Further inquiries can be directed to the corresponding author.

## Ethics Statement

Written informed consent was obtained from the patient for the publication of any potentially identifiable images or data included in this article.

## Author Contributions

JD conceived the idea of the case report. JD, YC, and YG were involved in the patient’s clinical management. JD performed the literature search and drafted the manuscript. YC designed and supervised the study. All authors contributed to the article and approved the submitted version.

## Funding

This work was supported by the Natural Science Foundation of Jiangxi Province, No. 20171BAB205057.

## Conflict of Interest

The authors declare that the research was conducted in the absence of any commercial or financial relationships that could be construed as a potential conflict of interest.

## Publisher’s Note

All claims expressed in this article are solely those of the authors and do not necessarily represent those of their affiliated organizations, or those of the publisher, the editors and the reviewers. Any product that may be evaluated in this article, or claim that may be made by its manufacturer, is not guaranteed or endorsed by the publisher.
